# Localization Microscopy Analyses of MRE11 Clusters in 3D-Conserved Cell Nuclei of Different Cell Lines

**DOI:** 10.3390/cancers10010025

**Published:** 2018-01-22

**Authors:** Marion Eryilmaz, Eberhard Schmitt, Matthias Krufczik, Franziska Theda, Jin-Ho Lee, Christoph Cremer, Felix Bestvater, Wladimir Schaufler, Michael Hausmann, Georg Hildenbrand

**Affiliations:** 1 Kirchhoff-Institute for Physics, University of Heidelberg, Im Neuenheimer Feld 227, 69120 Heidelberg, Germany; Marion.Eryilmaz@medma.uni-heidelberg.de (M.E.); eschmitt@kip.uni-heidelberg.de (E.S.); krufczik@kip.uni-heidelberg.de (M.K.); f.theda@gmx.de (F.T.); jin-ho.lee@kip.uni-heidelberg.de (J.-H.L.); hilden@kip.uni-heidelberg.de (G.H.); 2 Institute of Molecular Biology, Ackermannweg 4, 55128 Mainz, Germany; C.Cremer@imb-mainz.de; 3 German Cancer Research Center (DKFZ), Im Neuenheimer Feld 280, 69120 Heidelberg, Germany; f.bestvater@dkfz.de (F.B.); w.schaufler@dkfz.de (W.S.); 4 Department Radiation Oncology, Universitätsmedizin Mannheim, University of Heidelberg, Theodor-Kutzer-Ufer 3-5, 68159 Mannheim, Germany

**Keywords:** single-molecule localization microscopy, nano-distance analysis, MRE11 repair foci, DNA damaging, DNA repair, H2AX phosphorylation, γH2AX formation

## Abstract

In radiation biophysics, it is a subject of nowadays research to investigate DNA strand break repair in detail after damage induction by ionizing radiation. It is a subject of debate as to what makes up the cell’s decision to use a certain repair pathway and how the repair machinery recruited in repair foci is spatially and temporarily organized. Single-molecule localization microscopy (SMLM) allows super-resolution analysis by precise localization of single fluorescent molecule tags, resulting in nuclear structure analysis with a spatial resolution in the 10 nm regime. Here, we used SMLM to study MRE11 foci. MRE11 is one of three proteins involved in the MRN-complex (MRE11-RAD50-NBS1 complex), a prominent DNA strand resection and broken end bridging component involved in homologous recombination repair (HRR) and alternative non-homologous end joining (a-NHEJ). We analyzed the spatial arrangements of antibody-labelled MRE11 proteins in the nuclei of a breast cancer and a skin fibroblast cell line along a time-course of repair (up to 48 h) after irradiation with a dose of 2 Gy. Different kinetics for cluster formation and relaxation were determined. Changes in the internal nano-scaled structure of the clusters were quantified and compared between the two cell types. The results indicate a cell type-dependent DNA damage response concerning MRE11 recruitment and cluster formation. The MRE11 data were compared to H2AX phosphorylation detected by γH2AX molecule distribution. These data suggested modulations of MRE11 signal frequencies that were not directly correlated to DNA damage induction. The application of SMLM in radiation biophysics offers new possibilities to investigate spatial foci organization after DNA damaging and during subsequent repair.

## 1. Introduction

When in the early 1930s Ernst Ruska and Max Knoll developed the first electron microscope [1], super-resolution started its triumph into biological and biomedical research. About 50 years later, the application of near-field optics for super-resolution [2,3,4] and developments of super-resolution far-field microscopy like 4Pi or STED (Stimulated Emission Depletion) microscopy [4,5,6,7,8] demonstrated the potentials of light microscopy for super-resolution applications. An attractive approach using nothing else than “standard” detection equipment was developed in the 1990s [8] as “Spectral Distance Precision Microscopy (SDPM)” [9] and applied to quantitative analyses of genome regions [10,11]. 

With the development of nowadays fluorescent dyes and multiple fluorescent proteins, it has become possible to separate single fluorescent tags of specific single-molecule labels. The potentials of the combination of instrumentation, specific specimen labelling strategies and appropriate fluorescent tags induced the developments of a variety of novel techniques such as, for instance PALM (Photo Activated Localization Microscopy), F-PALM (Fluorescence-PALM), STORM (Stochastic Optical Reconstruction Microscopy), RESOLFT (reversible saturable optical linear (fluorescence) transitions) microscopy, etc. (for review see [4,8]), which are summarized by “Single-Molecule Localization Microscopy (SMLM)” [8,12]. Since the preparation conditions of specimens for most SMLM techniques are very similar to those applied in standard fluorescence microscopy, SMLM offers broad perspectives of applications in many fields of biological and biomedical research as well as in diagnostics [13,14].

For the experiments described here, an embodiment of SMLM is used in which standard fluorescent dyes can be switched between spectral “on” and “off” states [15,16] to achieve temporal isolation and thus spatial separation of single-molecule signals. From a reversible dark state, the fluorescent molecules can randomly return to the emission state and emit their photons when irradiated by light. Each of the emitting fluorophores is represented by an Airy disc in the microscopic image. The centre-of-mass (barycentre) of such a disc approximates the location of the emitting molecule. This allows not only the precise determination of object positions but also the calculation of their spatial distances in the 10 nm regime [14,17,18]. Using the matrix of the coordinates of fluorescent tags, all acquired positions and distances of fluorescent molecules can be analysed without generation of an image and thus without mathematics of computer image processing [14]. From this matrix, an artificial “pointillist”, super-resolution image can also be prepared, in which the effective resolution is only depending on the localization precision [18]. 

Different applications have demonstrated the power of SMLM for biological and biomedical measurements, e.g., analyses of chromatin in cell nuclei [19,20,21], of chromatin loops [22], of proteins in cytoplasm [23] and membranes [24,25], to name a few. In biological and biomedical radiation research [26,27], radiation-induced conformational chromatin changes [28], γH2AX kinetics after irradiation or folate deficiency [14,29], Erb-receptor re-organization and spatial connexin modulations [30] as well as gold nano-particle incorporation kinetics and DNA damage response [31,32] have been analysed. 

DNA double-strand breaks are the most severe damage in the genome of a cell nucleus that can be caused by ionizing radiation. These damages cause an instant activation of the repair machinery with phosphorylation of H2AX as one of the very first chromatin modifications [33,34,35]. Depending on the cell cycle state, the functional activity of genes, the break position along the DNA sequence, the temporal state of DNA compaction, etc., cells have to decide about the choice of the repair pathway at the given damaged site of the genome [36]. Although the major players and their functions in the repair pathways have been investigated, the central mechanism leading to the final decision is not clearly understood up to now and therefore a subject of research and scientific debate. 

Homologous recombination repair (HRR) [37], a rather slow but error-free repair process, needs an intact DNA sequence template of the homologous chromosome along the complementary strand reconstructed. Non-homologous end joining (NHEJ), a very frequently used repair process, can cause errors but is fast compared to HRR. Several specific proteins process the broken DNA ends by resection and re-connect the broken double strand at appropriate base complements. These two repair procedures are the most often chosen pathways (for review see [38,39]). In repetitive DNA units, HRR is sometimes suppressed and single-strand annealing (SSA) takes place instead. In cases where NHEJ fails, for instance, at higher doses (>2 Gy), an alternative NHEJ process (a-NHEJ) can occur, which is a slow and error-prone repair process [39,40]. 

Each of these repair processes requires a defined protein arrangement for DNA strand end processing, end joining or sequence repair [41]. A better understanding of the repair pathways and the proteins involved would promote individualized radiation cancer therapy by using small molecules for inhibition of repair [42,43]. Although many steps of DNA strand resection and processing, protein recruitment sequence and kinetics, or repair protein function and interaction with DNA have been investigated in detail, the question as to what makes up the cell’s decision for a certain pathway is still insufficiently answered. Physical as well as topological parameters of the DNA strand break environment may also contribute to the repair pathway choice as epigenetic conditions and cellular functioning or differentiation may do [26,27]. This has reasoned several electron and light microscopic studies to elucidate the spatio-temporal organization of repair foci with high even molecular resolution [44,45,46,47,48,49,50].

At that point, the genome architecture and the architecture of embedded repair complexes on the micro- and especially on the nano-scale become important in general in order to study mechanistic effects based on the molecular arrangements. This opens new perspectives in the application of SMLM in biological radiation research [14,27]. This has also reasoned our study. 

In the following, we will show analyses of the protein MRE11 [51,52] arrangement in nuclei of two different cell lines (breast cancer cell line MCF-7, skin fibroblast cell line CCD-1059SK) after low LET irradiation with a dose of 2 Gy and during a time course of repair of up to 48 h. MRE11 together with RAD50 and NBS1 form the MRN complex [53] by co-localizing at the DNA double-strand break sites. Together with other protein complexes like CtIP, MRE11 is involved in strand end resection in HRR and a-NHEJ [52,54,55] and forms distinct foci embedded in a γH2AX environment as being visualized by conventional fluorescence microscopy using specific antibodies [56]. The foci are resolved in a topology of single molecules by SMLM. Based on distance measurements, MRE11 molecular arrangements and dynamics are studied and compared between the two cell lines after irradiation in comparison to non-irradiation conditions. These data will be also seen in relation to γH2AX molecular arrangements at the same time points. 

## 2. Materials and Methods 

### 2.1. Cell Culture and Specimen Preparation 

The human breast cancer cell line MCF-7 was established from a pleural effusion of a 69-year-old female. The cells have an aneuploidy karyotype with stable MRE11 overexpression (clinically associated with malignant breast cancer) [57]. The human skin fibroblast cell line CCD-1059SK was established from a biopsy of a 20-year-old female. Both cell lines grow adherently. 

MCF-7 cells were grown in RPMI medium with 10% FCS (fetal calf serum) and 1% penicillin-streptomycin. The CCD-1059SK cells were grown in Eagle’s Minimum Essential medium (EMEM) with 10% FCS, 1% L-glutamine, 1% HEPES and 1% penicillin-streptomycin. The cells were growing in a humidified incubator at 37 °C and with 5% CO_2_. For SMLM, cells were seeded on 10 × 10 mm^2^ cover slips: To circumvent contamination of the cells, the cover slips were sterilized at 121 °C for 20 min. The coverslips were then transferred into Petri dishes with four internal wells. The cells were seeded at a given density determined by a Neubauer chamber (for MCF-7: 50,000 cells; for CCD-1059SK fibroblasts: 80,000 cells per dish) and further incubated for 48 h in a humidified incubator at 37 °C and 5% CO_2_. 

Cells were irradiated with the linear accelerator Artriste (Siemens, Erlangen, Germany) using 6 MV photon energy at a radiation dose of 2 Gy (dose rate 3 Gy/min). Afterwards, the cells were further cultivated. At defined time points (10 min, 30 min, 60 min, 120 min, 180 min, 24 h and 48 h) after irradiation, the medium was removed and the cells were washed with 5 mL 1× DPBS (Dulbecco´s phosphate-buffered saline) twice and incubated in 3.7% formaldehyde (prepared freshly from paraformaldehyde) for 10 min. The formaldehyde was removed by washing the cells twice in DPBS for 5 min. In parallel to the irradiated cells, the same procedure was performed on non-irradiated control cells subjected to the same time course. Finally, cells were stored in 1x DPBS and 0.1% NaN_3_ at 4 °C until further use.

### 2.2. Immunohistochemistry and Specimen Preparation for Single-Molecule Localization Microscopy 

For antibody labelling, the cells were washed for 5 min in 1x DPBS twice in order to remove NaN_3_. Then the cells were permeabilized by 0.2% Triton X-100 for 6 min at room temperature (RT). The cells were washed in 1× DPBS for 5 min at RT twice and incubated in 2% bovine serum albumin (BSA) on a shaker for 60 min at RT. In the next step, the primary rabbit anti-MRE11 antibodies (Abcam, Berlin, Germany) were dripped on parafilm in a 1:800 dilution and the cover slip with the cells was placed in the antibody solution. The cover slip was incubated in a humidified chamber for 2 h at RT and then overnight at 4 °C. The next day, the cells were rinsed with 0.2% Triton X-100 and washed with 1x DPBS for 5 min twice at RT. A secondary AlexaFluor 488 goat anti-rabbit antibody (Invitrogen, Thermo Fisher Scientific, Schwerte, Germany) was put on parafilm in a dilution of 1:250 and the cover slip with the cells was placed in the antibody solution and incubated in a humidified chamber for 45 min at 37 °C. From then on, light exposure to the fluorescent labelled specimen was avoided. In some experiments, a mouse anti-γH2AX antibody (Merck, Darmstadt, Germany) was used in addition, labelled by a secondary AlexaFluor 568 goat anti-mouse antibody (Invitrogen). 

After incubation, the cells were washed in 1x DPBS for 5 min twice. For cross-linking the cells were incubated in 3.7% formaldehyde for 5 min followed by washing in 1x DPBS for 5 min twice. The cell nuclei were counterstained in DAPI (4′,6-Diamidin-2-phenylindol; 1 μL in 20 mL DPBS) for 5 min at RT followed by washing in 1x DPBS for 5 min twice. Finally, the cover slips were air dried and the cell grown side positioned on a cleaned microscope slide with 15 μL ProLong^®^ Gold (Thermo Fisher Scientific, Schwerte, Germany) as antifade embedding medium. The cover slips were sealed with nail polish, ProLong^®^ Gold polymerized for 24 h at 4 °C before localization microscopy. The quality of immunohistochemistry staining was checked by conventional fluorescence microscopy.

### 2.3. Single-Molecule Localization Microscopy 

**Figure 1:**
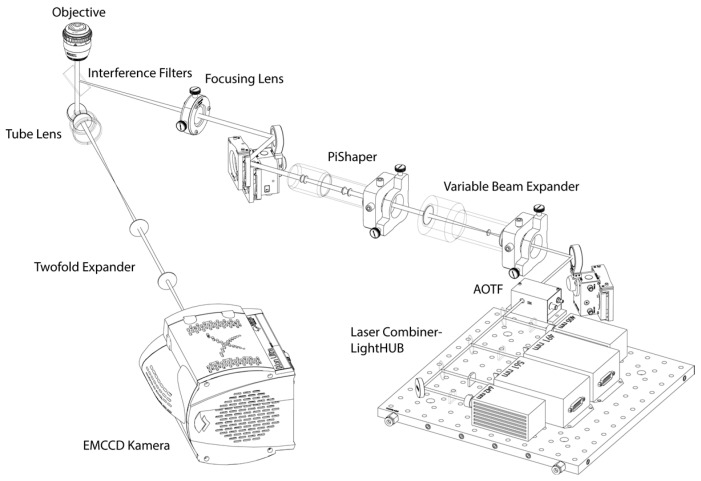
Schematic illustration of the localization microscope set-up.

SMLM was performed using a specially manufactured localization microscope (Figure 1) with enhanced thermomechanical stability. By indoor climate regulation and separate cooling of optical elements, the instrumental temperature was maintained in the range of ±10 mK. During a time course of 2 min image acquisition time, the mechanical drift of the specimen was less than 10 nm.

The illumination light path was equipped with a LightHub—laser combiner (Omicron Laserprodukte GmbH, Rodgau-Dudenhofen, Germany) assembled with four laser lines (405 nm, 491 nm, 561 nm, 642 nm), a polychromatic AOTF (Acousto Optical Tunable Filter; AA Opto Electronic, Orsay CEDEX, France), a variable beam expander 10BE03-2-8 (Standa Ltd., Vilnius, Lithuania) and a Flat-Top-Profile forming optics—PiShaper (AdlOptica GmbH, Berlin, Germany). The circular Flat-Top laser beam profile was projected (downscaled) into the object plane using an achromatic focusing lens (f = 250 mm) and a 100×/NA 1.46 oil plan apochromatic objective lens (Carl Zeiss Microscopy, Göttingen, Germany). For the AlexaFluor 488 dye and for the AlexaFluor 568 dye used here, the illumination wavelength of 491 nm and 561 nm, respectively, was used resulting in about 1 kW/cm^2^ power density in the object plane. The fluorescence light in the detection path is separated from the illumination light using two quadband interference filter glasses F73-410 and F72-866 (AHF analysentechnik AG, Tübingen, Germany) and is projected (magnified) by the objective—tube lenses pair (Carl Zeiss Microscopy, Göttingen, Germany) and an additional twofold expander on the Andor Ultra EMCCD (Andor Technology, Belfast, Northern Ireland). All images were acquired after a 2 h start-up phase for thermal stabilization. For each cell nucleus, a time stack of 2000 image frames with an integration time of 100 ms each was registered and saved in 16-bit grey-scale TIFF image stack format. For each cell line and repair/cultivation time point, 20 cell nuclei were recorded and evaluated.

### 2.4. Data Evaluation

For the determination of the local positions of the detected dye molecules from the blinking events, an algorithm described in [58] was applied that is based on the subtraction of the brightness values of two successive frames. This method enables the differentiation of the blinking events from the background. For each detected blinking point, the program defines a two-dimensional Gaussian distribution depending on the signal position and localization precision. A so-called “Orte-Matrix” was produced which contains information about signal amplitude, the lateral x- and y-coordinates, the standard deviations in x and y direction, position errors, etc. [14,15,22,59]. From this matrix, pointillist images of the loci spread artificially by the localization precision can be produced. It is also possible to encode the point brightness by the number of neighbors in a given surrounding. This is called nearest-neighbor image.

For the identification of clusters [14,25], appropriate parameters were iteratively determined. By visual inspection and comparison of thresholds for 100–120 points minimum per focus, MRE11 and γH2AX clusters were calculated to a minimum radius of 200 nm and a minimum number of 60 molecules (MRE11) or 110 molecules (γH2AX) per cluster. These sizes correlate to foci sizes obtained by conventional microscopy [60].

## 3. Results and Discussion

MRE11 foci were analyzed to obtain further insights into the assembling of the proteins which undergo multiple functional processes accompanied by homo-dimerization under certain conditions. After exposure to ionizing low LET radiation of 2 Gy dose, the spatial distribution of MRE11 was analyzed in cell nuclei at certain time points during repair. At these time points, aliquots of the same cell population were fixed and subjected to localization microscopy. For comparison, aliquots of control cells were fixed at the same time points. These cells were subjected to the same cultivation and preparation steps without radiation exposure.

After radiation-induced DNA damaging, MRE11 as a member of the MRN complex is primarily involved in HRR and a-NHEJ of DNA double-strand breaks [51], but the MRN complex is not only a prominent actor in radiation response. In meiotic and mitotic homology-driven repair, the MRN complex influences double-strand break repair structurally by forming bridges between DNA molecules and enzymatically promoting resection of double-strand break ends [61]. Recent studies suggest that MRN is also responsible for double-strand break clustering [62]. Moreover, MRE11 is involved in telomere homeostasis, apoptosis and immune response. This has reasoned the choice of two different cell lines: (a) MCF-7, a breast cancer cell line with a stable MRE11 overexpression without any radiation treatment; (b) CCD-1059SK, a skin fibroblast cell line with a normal MRE11 expression without radiation treatment.

**Figure 2:**
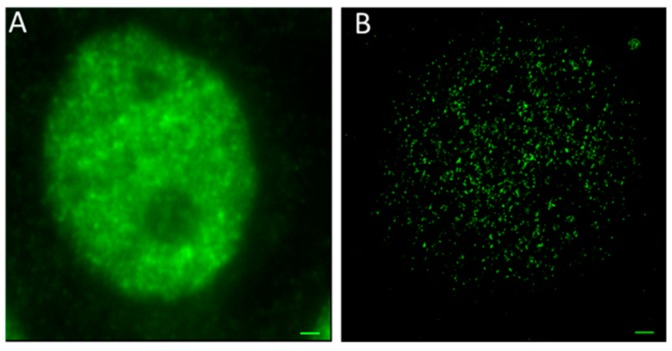
Fluorescence images of MCF-7 cell nuclei after specific labelling of MRE11 by antibodies. (**A**) conventional wide-field image; (**B**) pointillist single-molecule localization microscopy (SMLM) image obtained from the “Orte-Matrix” (scale bar 1 μm). Typically, about 50,000 points could be separated. Depending on the MRE11 activation state, a “network” like organization of molecules with areas of different concentration was recorded.

In order to show a visual impression of the differences in resolution, Figure 2 shows two fluorescence images of cell nuclei of MCF-7 after specific antibody labelling of MRE11. In the wide-field image, the fluorescence of all labelling molecules is blurred and spread over the whole cell nucleus due to diffraction and thus limited resolution of the objective lens. In contrast, the localization image has been produced as an artificial image obtained from the matrix of the coordinates of detected blinking events (“Orte-Matrix”). The resulting pointillist image precisely resolves the individual loci of MRE11 molecules and indicates their in-homogenous distribution in the cell nucleus. A comparison of both images (conventional microscopy and SMLM) shows the resolution improvement. Quantification of the molecule topology allows for improved insights into the MRE11 organization.

**Figure 3:**
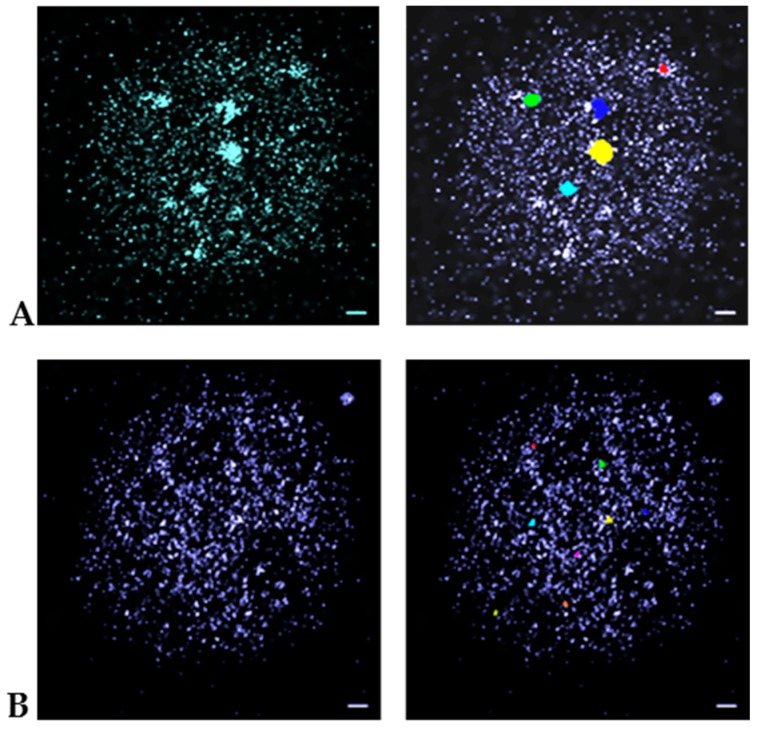
Pointillist SMLM images obtained from the “Orte-Matrix” of MCF-7 cell nuclei. (**A**) γH2AX labelling; (**B**) MRE11 labelling. The left images are nearest-neighbor illustrations indicating foci by increased intensity. The right images show the clusters as identified by the algorithm applied. These images reveal differently shaped clusters of at least a minimum number of points (for γH2AX: 110 points; for MRE 11: 60 points) in a given surrounding (for γH2AX: radius of 200 nm; for MRE11: radius of 100 nm). In general, the MRE11 clusters are considerably smaller than the γH2Ax-clusters. Scale bar 1 μm. Note: The different colors of the clusters are only used to better separate them for visualization.

In Figure 3, examples for the cluster identification process from SMLM measurements are given. Using the nearest-neighbor image visualization gives an impression of intensity foci compatible to wide-field imaging. The application of the clustering algorithm highlights differently shaped MRE11 clusters containing at least 60 points in a 100 nm surrounding. Compared to γH2AX clusters (minimum 110 points in a 200 nm surrounding) they were considerably smaller.

**Figure 4:**
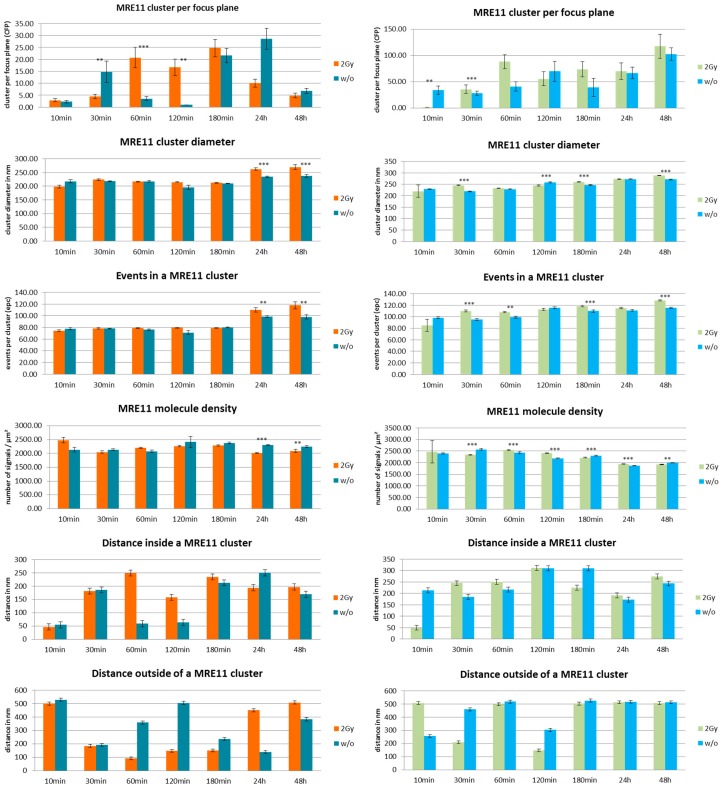
Overview of the results obtained from SMLM measurements. Left panels show the data obtained from MCF-7 breast cancer cell nuclei; right panels show the data obtained from cell nuclei of CCD-1059SK fibroblasts. The columns of each panel represent the mean values calculated from 20 nuclei each. The error bars on top of the column indicate the standard deviation. For each time step after the irradiation process, the data are given for cells exposed to 2 Gy low LET ionizing radiation (“2 Gy”) and for cells subjected to the same culturing procedure but not to radiation treatment (“w/o”). Level of significance between the corresponding values for “2 Gy” and “w/o”: *** = 0.1%; ** = 1%. For further details see text.

**Figure 5:**
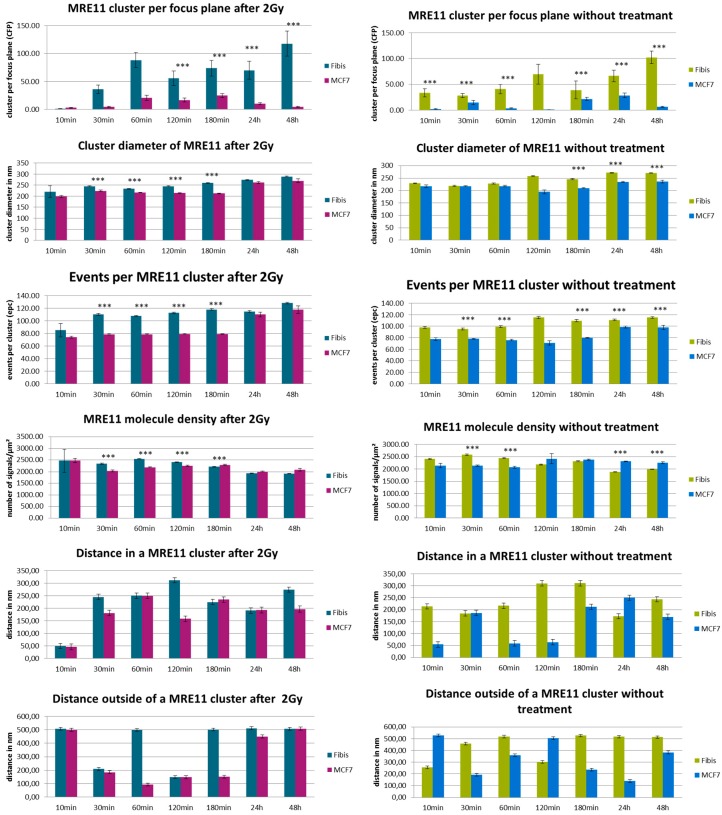
Overview of the results obtained from SMLM measurements. Left panels show the data obtained after radiation exposure for MCF-7 breast cancer cell nuclei (“MCF-7”) in comparison to cell nuclei of CCD-1059SK fibroblasts (“Fibis”); right panels show the data obtained without radiation treatment. The columns of each panel represent the mean values calculated from 20 nuclei each. The error bars on top of the column indicate the standard deviation. For each time step after the irradiation process, the data are given for cells exposed to 2 Gy ionizing radiation and for cells subjected to the same culturing procedure but not to radiation treatment. Level of significance between the corresponding values: *** = 0.1%. For further details see text.

In Figure 4, a series of histograms represents an overview over the measured data for the different specimens of MCF-7 cells (left panels) and CCD-1059SK fibroblasts (right panels). The columns of the histograms show the measured values for different times after the irradiation treatments for specimens exposed to a dose of 2 Gy in comparison to the non-treated control specimens (referred as “w/o”). In Figure 5, the comparison of the two cell lines are shown for the different treatments (2 Gy radiation exposure left panels; without radiation treatment right panels).

In general, MRE11 formed more clusters in fibroblasts than in MCF-7 cells, which may be surprising since MCF-7 should have an overexpression of MRE11. After irradiation, the MRE11 proteins are recruited and at 60 min the number of clusters reached the maximum which was maintained until 180 min. The fibroblasts already showed a significantly higher level at 30 min for irradiated cells as compared to non-irradiated cells. This was not observed for MCF-7. In fibroblasts, the cluster formation seemed to be maintained until 48 h (end of our observation) without any reduction. In contrast, MRE11 cluster frequency is fluctuating in non-irradiated cells. This fluctuation is more pronounced in MCF-7 cells where the maximum occurs at 30 min and 24 h. The latter may be also due to the overexpression of MRE11 in MCF-7 cells in general. This might also explain the increase at 3 h and 24 h, especially with respect to the fact that the cells were not synchronized. 

**Figure 6:**
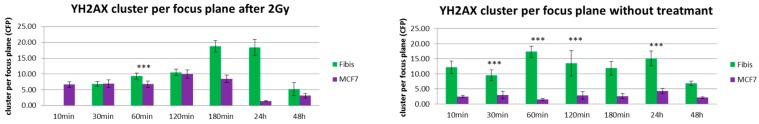
Number of detected γH2AX clusters in MCF-7 breast cancer cell nuclei (“MCF-7”) in comparison to cell nuclei of CCD-1059SK fibroblasts (“Fibis”). The columns of each panel represent the mean values calculated from 20 nuclei each. The error bars on top of the column indicate the standard deviation. For each time step after the irradiation process, the data are given for cells exposed to ionizing radiation of 2 Gy (**left**) and for cells subjected to the same culturing procedure but not to radiation treatment (**right**). Level of significance between the corresponding values: *** = 0.1%. For further details see text.

Since MCF-7 cells have a cell cycle of about 29 h or even less, these increases may be due to double strand break (DSB) repair induced during DNA replication in S-phase. The same reason depending on repair activity may explain the effect for fibroblasts. If the relative number of cells partly being synchronized may be less, MRE11 is on average more activated in this cell line. For comparison, the formation of γH2AX clusters was analyzed in both cell types. The number of γH2AX clusters did not exactly follow the fluctuations of MRE11 in the time course but showed a compatible behavior of increase and decrease (Figure 6). Again, the cluster number in fibroblasts is higher as compared to MCF-7 cells. Since non-irradiated fibroblasts show a similar fluctuating behavior, it might be considered that this increase in repair activity may reflect repair of spontaneous DSBs during DNA replication. In contrast to fibroblasts, MCF-7 cells only show γH2AX cluster formation on a background level (Figure 6, right panel).

**Figure 7:**
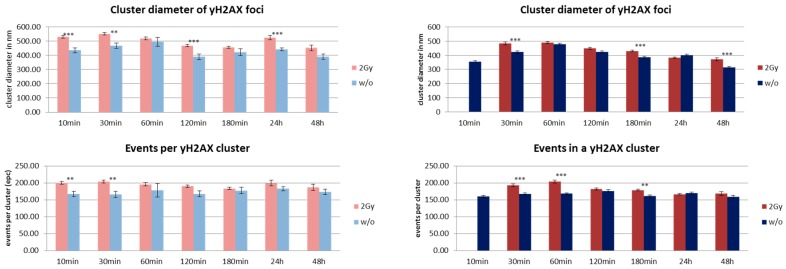
Overview of cluster size results obtained from SMLM measurements. **Left** panels show the data obtained from MCF-7 breast cancer cell nuclei; **right** panels show the data obtained from cell nuclei of CCD-1059SK fibroblasts. The columns of each panel represent the mean values calculated from 20 nuclei each. The error bars on top of the column indicate the standard deviation. For each time step after the irradiation process, the data are given for the cells exposed to 2 Gy low LET ionizing radiation (“2 Gy”) and for cells subjected to the same culturing procedure but not to radiation treatment (“w/o”). Level of significance between the corresponding values for “2 Gy” and “w/o”: *** = 0.1%; ** = 1%. For further details see text.

The MRE11 cluster diameters were always in the range of 200 to 250 nm, but in most cases the clusters were significantly larger in fibroblasts. The level of significance was better than 0.1% for 30–180 min after irradiation and for 3–48 h in the case of non-irradiated cells. The small distance variance indicated that the size of the clusters was very homogenous over all cell nuclei of the two lines. This was also true for non-irradiated cells, although the number of clusters as well as the number of labelled proteins was much lower. The MRE11 clusters were always smaller than the corresponding γH2AX clusters (Figure 7).

The number of MRE11 molecules (labelled events) in a cluster was in the range of 80 to 120 for both cell lines and types of treatment along the whole time course. However, fibroblasts showed a higher number of events. At most time points, this difference was significant on a 0.01% level. With the exception of the early recruitment phase (10 min), the number of molecules was equal or often higher for the irradiated specimens compared to the non-irradiated ones. In comparison, the number of tagged γH2AX molecules was higher for the corresponding clusters (Figure 7). The difference between MCF7 and fibroblasts was not significant.

**Figure 8:**
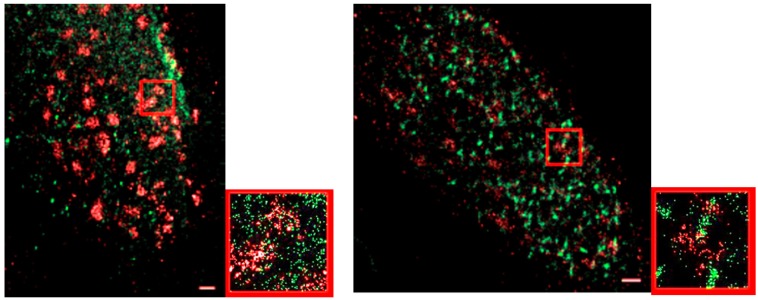
Two color SMLM images of two MCF-7 cell nuclei after irradiation. **Left** images show the situation 30 min post irradiation; **right** images 180 min post irradiation. While γ-H2AX (red labelling) has already formed foci after 30 min, MRE11 (green labelling) is still more dispersed. After 180 min, both signals show strong clustering (foci formation). The small images are enlarged sections of the larger images (red rectangle).

In literature, it was reported that MRE11 foci are detectable 30 min after radiation treatment in human fibroblasts [63]. This was supported by our study, although the number was further increasing afterwards. MCF-7 cells indicated a few foci at 30 min but in contrast to the control cells it was negligible. However, a strongly elevated number of clusters occurred after 60 min with the maximum number of clusters 180 min post irradiation. This typical behavior was verified in different independent experiments [64,65]. In some two-color experiments, it was shown that after 30 min, γ-H2AX has formed foci, while MRE11 is still being recruited, indicated by dispersed molecules. After 180 min, both repair complexes formed distinct clusters (Figure 8). These data show that SMLM measurements are also feasible between different color channels [35] and thus different proteins. Future investigations will show systematic studies of arrangements of repair type typical proteins in correlation to γH2AX tags (manuscript in preparation). 

After having formed a high amount of MRE11 clusters at 60 min, the irradiated MCF-7 cells showed an equal and often lower MRE11 molecule density in the irradiated specimens compared to the non-irradiated ones (Figure 4 and Figure 5). This was not the case for fibroblasts where the irradiated specimens showed a significantly higher molecule density. This may be due to the different MRE11 expression level. The distances between molecules that were determined to refer to a cluster followed the cluster formation behavior. After 60 min when the clusters were formed, the distances between the fluorescent events increased. This may indicate molecular activity inside the clusters, i.e., that due to additional proteins or molecular complexes recruited, additional space may be required between the labelled MRE11 molecules. Such distance increase between clustering proteins has been also observed for other molecules and complexes involved in cellular activities (see for instance [25,30]). This behavior was also observed to the non-irradiated cases where the distance size followed the fluctuating behavior of the cluster frequency. Looking on the distances between signals outside the clusters, the non-irradiated MCF-7 cells showed a strongly inverse behavior to the distances inside clusters. For the irradiated MCF-7 cells, this was only the case for the early repair phase until 180 min. At the later time points, the distances inside and outside clusters were increased. The fibroblasts did not show such a conclusive behavior of the distance values. More fluctuations seemed to occur.

## 4. Conclusions

DNA double-strand breaks induced by ionizing radiation cause an instant activation of the repair machinery. Homologous recombination repair (HRR) and non-homologous end joining (NHEJ), the most often chosen repair pathways, require defined protein arrangements for DNA strand end processing, end joining or sequence repair. Thus, it seems to be important to elucidate the local molecular topology of repair proteins at the different loci in the cell nucleus where repair may take place. By means of super-resolution localization microscopy, molecular arrangements in repair foci can be visualized and, by means of systematic measurements along a time course of repair, dynamics of proteins and protein interactions may be studied. Single-molecule localization microscopy measurements are a tool to better understand the contribution of topological arrangements to mechanism behind the repair and to figure out the spatiotemporal conditions that may exist for the cell’s decision to use a certain repair pathway at a given damaged DNA side. 

Here, we demonstrated the potentials of SMLM for applications towards investigations of the internal organization and architecture of repair foci. This study has only been presented for just one important repair protein, MRE1 of the NRM complex, but opens perspectives in the application of SMLM in biological radiation research. Future investigations on other repair foci and combinations of proteins within individual foci will be performed. Together with measurements of chromatin allocation inside the foci, such experiments may give a conclusive interpretation of the topology inside the foci and the functional interactions in the repair machinery.
